# Real-Time PCR Cycle Threshold Values for the BRAF^V600E^ Mutation in Papillary Thyroid Microcarcinoma May Be Associated With Central Lymph Node Metastasis: A Retrospective Study

**DOI:** 10.1097/MD.0000000000001149

**Published:** 2015-07-17

**Authors:** Vivian Y. Park, Eun-Kyung Kim, Hye Sun Lee, Hee Jung Moon, Jung Hyun Yoon, Jin Young Kwak

**Affiliations:** From the Department of Radiology, Research Institute of Radiological Science, Severance Hospital (VYP, E-KK, HJM, JHY, JYK); and Biostatistics Collaboration Unit, Medical Research Center, Yonsei University, College of Medicine, Seoul, Korea (HSL).

## Abstract

Papillary thyroid microcarcinoma (PTMC) usually has excellent prognosis, but a small subset shows aggressive behavior. Although the B-Raf proto-oncogene, serine/threonine kinase (BRAF)^V600E^ mutation is the most common oncogenic alteration in PTMCs, it is frequently heterogeneously distributed within tumors. The aim of this study was to investigate the association of the BRAF^V600E^ mutation found in fine needle aspirates from PTMCs with known clinicopathologic prognostic factors, based on both its presence and a quantitative approach that uses cycle threshold (Ct) values obtained by a real-time PCR technique.

The 460 PTMC patients were included, with 367 patients having the BRAF^V600E^ mutation. Clinicopathologic variables were compared between patients with and without the BRAF^V600E^ mutation. BRAF^V600E^ Ct values were compared according to clinicopathologic prognostic factors. Multivariate analyses were performed to evaluate factors predicting extrathyroidal extension and central and lateral lymph node metastasis (LNM). Each analysis used either the BRAF^V600E^ mutation status or the Ct value as an independent variable for all the study patients and the 367 BRAF^V600E^-positive patients. Receiver-operating characteristic (ROC) curve analysis was performed to evaluate the diagnostic performance of BRAF^V600E^ Ct values in predicting central and lateral LNM.

The BRAF^V600E^ mutation status was not associated with clinicopathologic prognostic factors among the 460 PTMC patients. Of the 367 BRAF^V600E^-positive patients, Ct values were significantly lower in patients with central and lateral LNM (*P* < 0.001, *P* = 0.007). The Ct value was the only independent factor to predict central LNM (OR 0.918, *P* = 0.025). The area under the ROC curve (AUC) for diagnosing central LNM was 0.623 (sensitivity, 50.0%; specificity, 71.9%) and for diagnosing lateral LNM, it was 0.796 (sensitivity, 71.4%; specificity, 94.7%).

In conclusion, real-time PCR Ct values for the BRAF^V600E^ mutation obtained from fine needle aspirates can be associated with central LNM in PTMC patients. Although BRAF^V600E^ Ct values did not reach statistical significance for predicting lateral LNM in our study, further validation through larger studies can be used to overcome any possible type-II errors. With further studies, Ct values for the BRAF^V600E^ mutation obtained from fine needle aspirates may have important implications for predicting both central and lateral LNM in patients with PTMCs.

## INTRODUCTION

Papillary thyroid carcinoma (PTC) accounts for approximately 90% of thyroid malignancies, with an increasing number of small thyroid cancers recently being detected due to the widespread use of ultrasonography (US) and US-guided fine-needle aspiration (US-FNA).^1,2^ Papillary thyroid microcarcinoma (PTMC) is defined as PTC that measures ≤10 mm in its greatest dimension, and the majority of PTMCs are indolent cancers that show favorable prognosis.^3^ Although various guidelines currently do not recommend FNA for subcentimeter nodules detected in patients without high-risk clinical features, the incidence of PTMC is increasing globally, from 6.1% to 29% of all thyroid cancers to 21.7% to 47%.^2,4,5^ However, a small but significant portion of PTMCs show aggressive behavior, with a 3.3% average rate of recurrence and a 0.5% mortality rate from PTMC.^3,5,6^ Thus, this aggressive subset would benefit from adjunctive tools that would enable its differentiation from the vast majority of indolent PTMCs.

The B-Raf proto-oncogene, serine/threonine kinase (BRAF) mutation is the most common oncogenic alteration detected in patients with PTC, with more than 90% characterized by the change of valine to glutamic acid at codon 600, a mutation designated as BRAF^V600E^.^7^ Its reported incidence varies from 28.2% to 95.2% of PTMCs, and a greater prevalence has been observed in the Korean population.^8–10^ Research on its association with clinicopathologic prognostic factors of PTMC has revealed conflicting results regarding bilaterality, multifocality, extrathyroidal invasion, and lymph node metastasis (LNM), in which studies were based on the presence or absence of the BRAF^V600E^ mutation and did not contain quantitative information.^8,10–13^ Clonal BRAF^V600E^ mutation is a rare occurrence and PTCs frequently consist of a mixture of tumor cells with wild-type and mutant BRAF.^14,15^ To address this topic, quantitative measurements of the BRAF^V600E^ mutation from surgically resected PTC specimens have been utilized and the BRAF^V600E^ mutation has been associated with tumor size, extrathyroidal invasion, and disease recurrence.^16–18^ However, to our knowledge, there has been no published study utilizing BRAF^V600E^ mutation testing in fine needle aspirates and none quantitatively analyzing the BRAF^V600E^ mutation in PTMC.

Therefore, the aim of this study was to investigate the association between the BRAF^V600E^ mutation found in fine needle aspirates of PTMCs and known clinicopathologic prognostic factors in a BRAF^V600E^-prevalent population, based on both the presence of the BRAF^V600E^ mutation and a quantitative approach that uses cycle threshold (Ct) values obtained by a highly sensitive real-time polymerase chain reaction (PCR) technique.

## MATERIAL AND METHODS

### Study Population

This study was approved by our institutional review board, and the requirement to obtain informed consent was waived. Between January 2011 and July 2012, 2390 patients were confirmed to have PTMC. Among them, 460 patients underwent US-FNA with simultaneous BRAF^V600E^ mutation testing using real-time PCR at our institution (a referral center). A total of 369 women (median age, 49 years; range, 19–76 years) and 91 men (median age, 43 years; range, 24–73 years) were included. Of them, 367 patients had the BRAF^V600E^ mutation confirmed through real-time PCR testing from fine needle aspirates. Twelve and 17 nodules were previously included in 2 prior studies, respectively. One study focused on the diagnostic performances of cytology, US, and BRAF^V600E^ mutation testing, and the other study focused on the additional value of the BRAF^V600E^ mutation analysis in thyroid nodules with “suspicious for malignant” cytology-lacking suspicious US features.^7,19^

### US-FNA and the BRAF^V600E^ Mutation Analysis

At our institution, US-FNA was performed with a 23-gauge needle attached to a 2-mL disposable plastic syringe using the freehand technique. FNAs were performed on either thyroid nodules with suspicious US features or the largest nodule if there were no suspicious US features. Each lesion was aspirated at least twice. Materials obtained from the aspiration biopsy were expelled onto glass slides and smeared. The material remaining in the syringe after cytological preparation was collected for the BRAF^V600E^ mutation analysis, which was performed at the request of the referring physician.

Real-time PCR was performed using the Applied Biosystems 7500 real-time PCR system (Applied Biosystems, Foster City, CA) under the following cycle conditions; denaturation at 50°C for 2 minutes (1 cycle), 95°C for 10 minutes (1 cycle), and 95°C for 15 seconds (1 cycle), followed by one step of annealing and elongation at 62°C for 45 seconds (40 cycles). A Real-Q BRAF^V600E^ Detection Kit (BioSewoom, Korea) was used for the PCR reactions. This is a ready-to-use kit that detects the BRAF^V600E^ (1799T > A) somatic mutation in the BRAF oncogene in a background of wild-type genomic DNA using a multiplex real-time PCR assay based on the TaqMan MGB probe system. BRAF^V600E^ amplification was detected by measuring the VIC fluorescence in the AB 7500 system. The internal control assay, labeled with 6-Carboxyfluorescein, was used to check for nucleic acid isolation and possible PCR inhibition. A region of exon 8 of the BRAF gene was used for amplification as the internal control. The 242 base pairs of the partial BRAF gene containing the V600E region was amplified from the human melanoma cell line, SK-MEL-28 (ATCC, Manassas, VA) with the BRAF^V600E^ mutation, and was inserted into the pZEM-T Easy Vector (Promega, Madison, WI) to produce the BRAF^V600E^ plasmid DNA. Analytical sensitivity was evaluated using the BRAF^V600E^ mutation plasmid clone and the 95% positive cut-off value (limit of detection) was calculated as 21.5 copy/μL by Probit analysis.^20^

The Ct was defined as the number of amplification cycles at which the level of fluorescent signal exceeded the threshold for the presence of the BRAF mutation. The cut-off value for a BRAF^V600E^ mutation-positive result was set to Ct 40, according to our previous research.^20^ This cut-off value was determined based on the average Ct value found through 100 repeats of low-positive concentrations of the BRAF^V600E^ plasmid DNA, for which a positive rate of 100% was achieved. The Ct value was determined from a log-linear plot of the PCR signal versus the cycle number, and was inversely related to the BRAF^V600E^ mRNA level. Thus, a low Ct value corresponds to a higher mRNA level.^16,21^

### Surgical Procedure

Total or near-total thyroidectomy was performed in patients who were either diagnosed or suspected of having multiple or bilateral tumors, extrathyroidal invasion or LNM based on preoperative assessment or intraoperative findings. Regardless of findings on physical examination or preoperative staging US, all patients underwent routine central compartment neck dissection including removal of the paratracheal, pretracheal, and prelaryngeal lymph nodes (LNs). Bilateral or ipsilateral central compartment neck dissection was each performed in patients who underwent total or near-total thyroidectomy or in those who underwent hemithyroidectomy, respectively. Lateral compartment neck dissection was selectively performed in patients diagnosed preoperatively as having lateral LNM by US-FNA. Intraoperative frozen biopsy was performed for LNs suspicious for metastases detected during surgery, but which were not found on preoperative staging US. If metastasis was confirmed, lateral compartments including levels 2, 3, 4 and anterior 5 were dissected.^22,23^

Of the 460 PTMC patients, 227 (49.3 %) patients underwent total or near-total thyroidectomy, 50 (10.9%) patients underwent subtotal thyroidectomy, and 183 (39.8%) patients underwent hemithyroidectomy. We evaluated tumor size, multifocality including both uni-and bilateral tumor foci, extrathyroidal extension, and presence of central and lateral LNM based on final surgical pathology reports.

### Statistical Analysis

Clinicopathologic characteristics of patients with and without the BRAF^V600E^ mutation were compared with the χ^2^ test or Fisher exact test for categorical variables and the Mann–Whitney U test for continuous variables. Multivariate logistic regression analysis was used to evaluate independent factors for extrathyroidal extension and central and lateral LNM including the BRAF^V600E^ mutation status, that is, the presence or absence of the BRAF^V600E^ mutation was analyzed as an independent variable.

For the 367 BRAF^V600E^-positive patients, BRAF^V600E^ Ct values were compared according to clinicopathologic prognostic factors using the Mann–Whitney U test. The Spearman correlation coefficient (*r*) was used to evaluate the association between the BRAF^V600E^ Ct values and continuous clinicopathologic variables (tumor size, patient age). We performed a separate multivariate logistic regression analysis to determine independent factors for extrathyroidal extension and central and lateral LNM. Odds ratios (ORs) with 95% confidence intervals (CIs) were calculated after the adjustment of all clinicopathologic factors. In addition, receiver-operating characteristic (ROC) curve analysis was performed to evaluate the diagnostic performance of BRAF^V600E^ Ct values in the prediction of central and lateral LNM.

A 2-tailed *P* value of less than 0.05 was defined as a statistically significant difference. Statistical analysis was performed with SPSS for Windows, version 20.0 (IBM Corporation, Armonk, NY) for all data analyses except the ROC curve analysis which was performed with SAS software (version 9.2, SAS Inc., Cary, NC).

## RESULTS

The median tumor size was 6 mm (range, 2–10 mm) for the 460 PTMCs (Table 1). We analyzed the association between the BRAF^V600E^ mutation status and variable clinicopathologic factors in the 460 PTMC patients who underwent BRAF^V600E^ mutation testing with real-time PCR. The BRAF^V600E^ mutation was detected in 79.8% (367/460) of the patients from preoperative fine needle aspirates. BRAF^V6006E^-positive patients were significantly older in age (*P* = 0.008), but none of the other clinicopathologic prognostic factors showed a significant difference between patients with and without the BRAF^V600E^ mutation (Table 1). On multivariate analysis, the BRAF^V600E^ mutation status was not significantly associated with any of the clinicopathologic prognostic factors (Table 2). Extrathyroidal extension was significantly associated with older age (OR 1.024, 95% CI 1.006–1.043, *P* = 0.011), female sex (OR 1.708, 95% CI 1.012–2.883, *P* = 0.045), and larger tumor size (OR 1.275, 95% CI 1.159–1.404, *P* < 0.001). Central LNM was significantly associated with younger age (OR 0.957, 95% CI 0.937–0.976, *P* < 0.001), larger tumor size (OR 1.152, 95% CI 1.039–1.279, *P* = 0.008), and multifocality (OR 1.649, 95% CI 1.021–2.665, *P* = 0.041). Lateral LNM did not show a significant association with any of the clinicopathologic-risk factors.

**TABLE 1:**
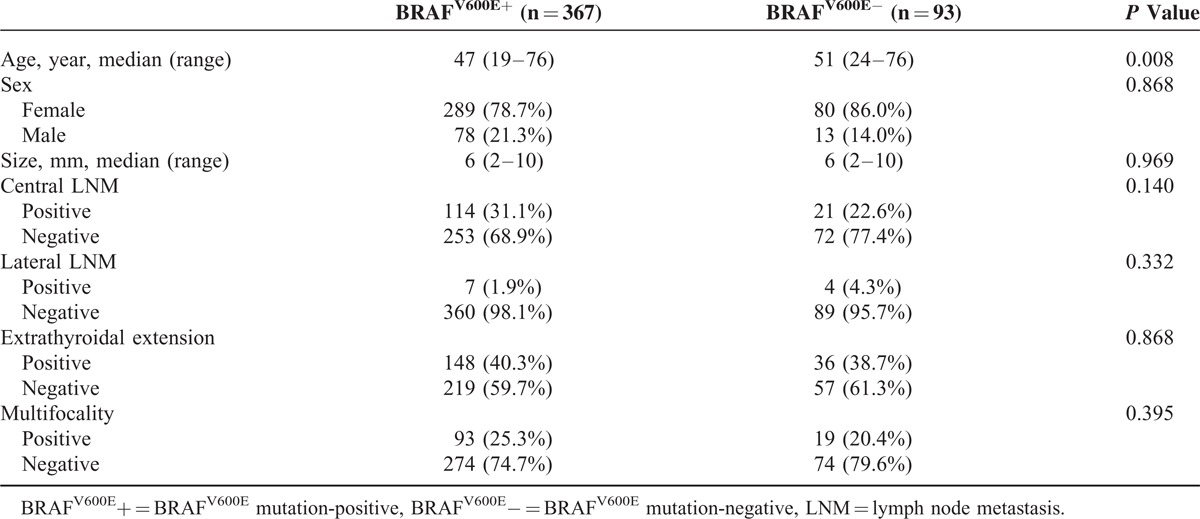
Clinicopathologic Characteristics and the BRAF^V600E^ Mutation Status in 460 Papillary Thyroid Microcarcinoma Patients

**TABLE 2:**
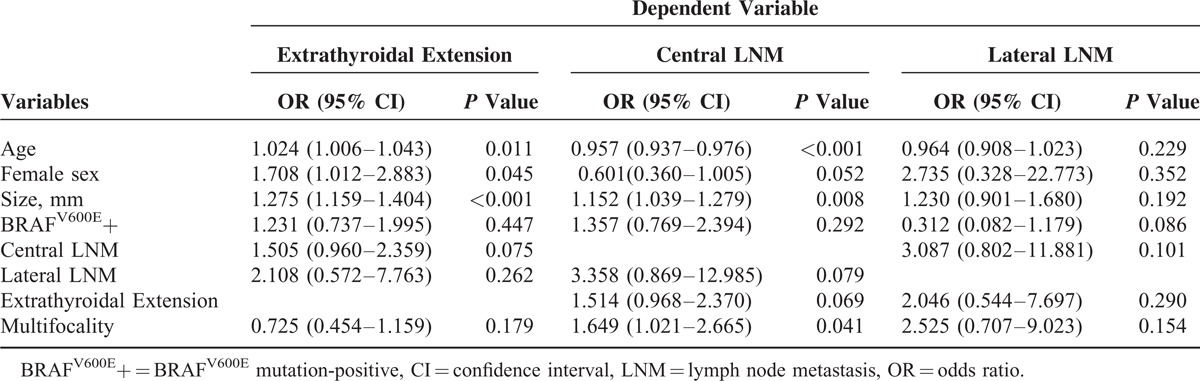
Multivariate Logistic Regression Analysis for Clinicopathologic Factors Based on the Presence or Absence of the BRAF^V600E^ Mutation in 460 Papillary Thyroid Microcarcinoma Patients

For the 367 PTMC patients with the BRAF^V600E^ mutation found through FNA, the association between Ct values of the BRAF^V600E^ mutation and variable clinicopathologic factors was analyzed. On univariate analysis, the quantitative expression of the BRAF^V600E^ mutation, expressed as a Ct value, was significantly lower in patients with central and lateral LNM (*P* < 0.001 and *P* = 0.007, respectively), but there was no significant difference according to sex, extrathyroidal extension, and multifocality (Table 3). BRAF^V600E^ Ct values were negatively correlated with tumor size, showing a weak but significant negative association (*r* = −0.306, *P* =  < 0.001). There was no significant association between BRAF^V600E^ Ct values and patient age (*r* = 0.022, *P* = 0.671). On multivariate analysis, the BRAF^V600E^ Ct value was the only independent factor to predict central LNM (OR 0.918, 95% CI 0.853–0.989, *P* = 0.025 (Table 4). Extrathyroidal extension was significantly associated with older age (OR 1.029, 95% CI 1.008–1.050, *P* = 0.006) and larger tumor size (OR 1.269, 95% CI 1.133–1.420, *P* < 0.001). Central LNM was also significantly associated with younger age (OR 0.963, 95% CI 0.942–0.985, *P* = 0.001) and larger tumor size (OR 1.139, 95% CI 1.011–1.282, *P* = 0.033). Lateral LNM did not show a significant association with any of the clinicopathologic-risk factors in the 367 BRAF^V600E^-positive PTMC patients (*P* = 0.111).

**TABLE 3:**
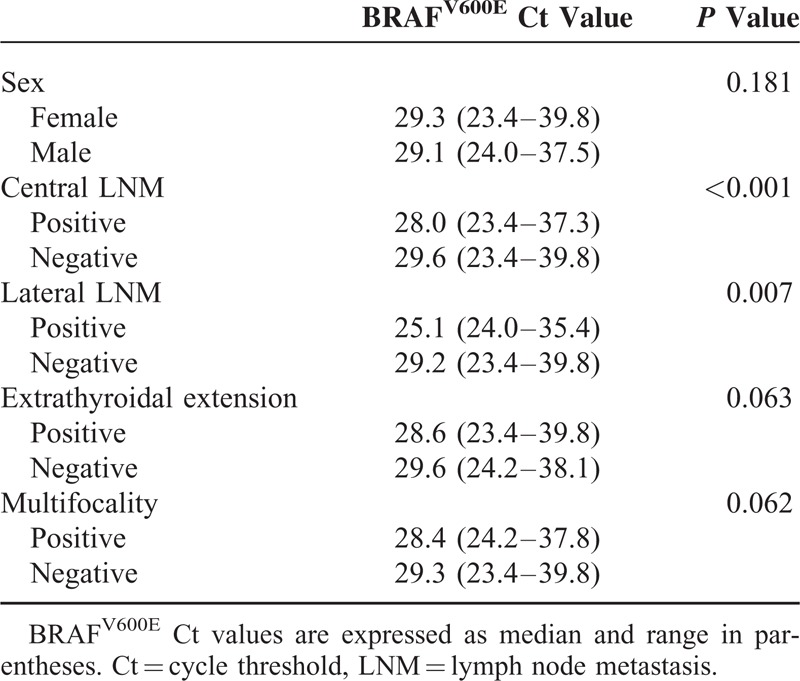
Clinicopathologic Characteristics and Quantitative Expression of the BRAF^V600E^ Mutation in 367 BRAF^V600E^-Positive Papillary Thyroid Microcarcinoma Patients

**TABLE 4:**
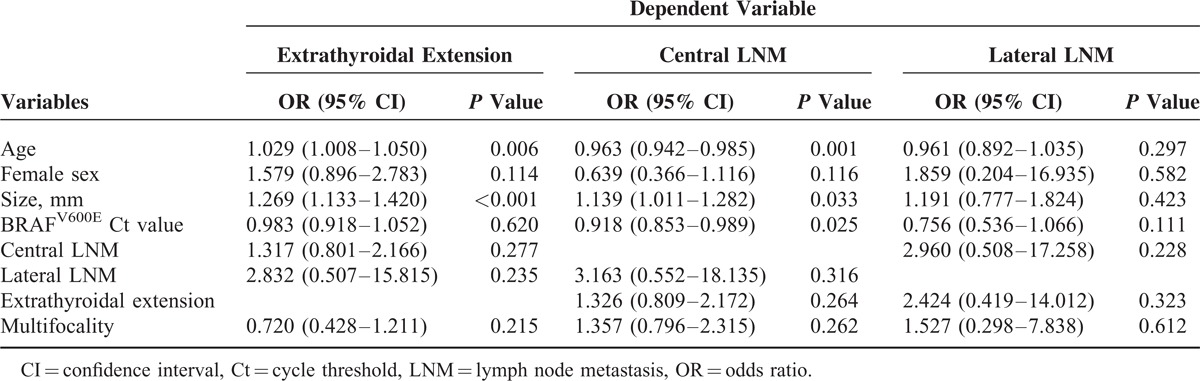
Multivariate Logistic Regression Analysis for Clinicopathologic Factors Based on the Quantitative Expression of the BRAF^V600E^ Mutation in 367 BRAF^V600E^-Positive Papillary Thyroid Microcarcinoma Patients

**FIGURE 1:**
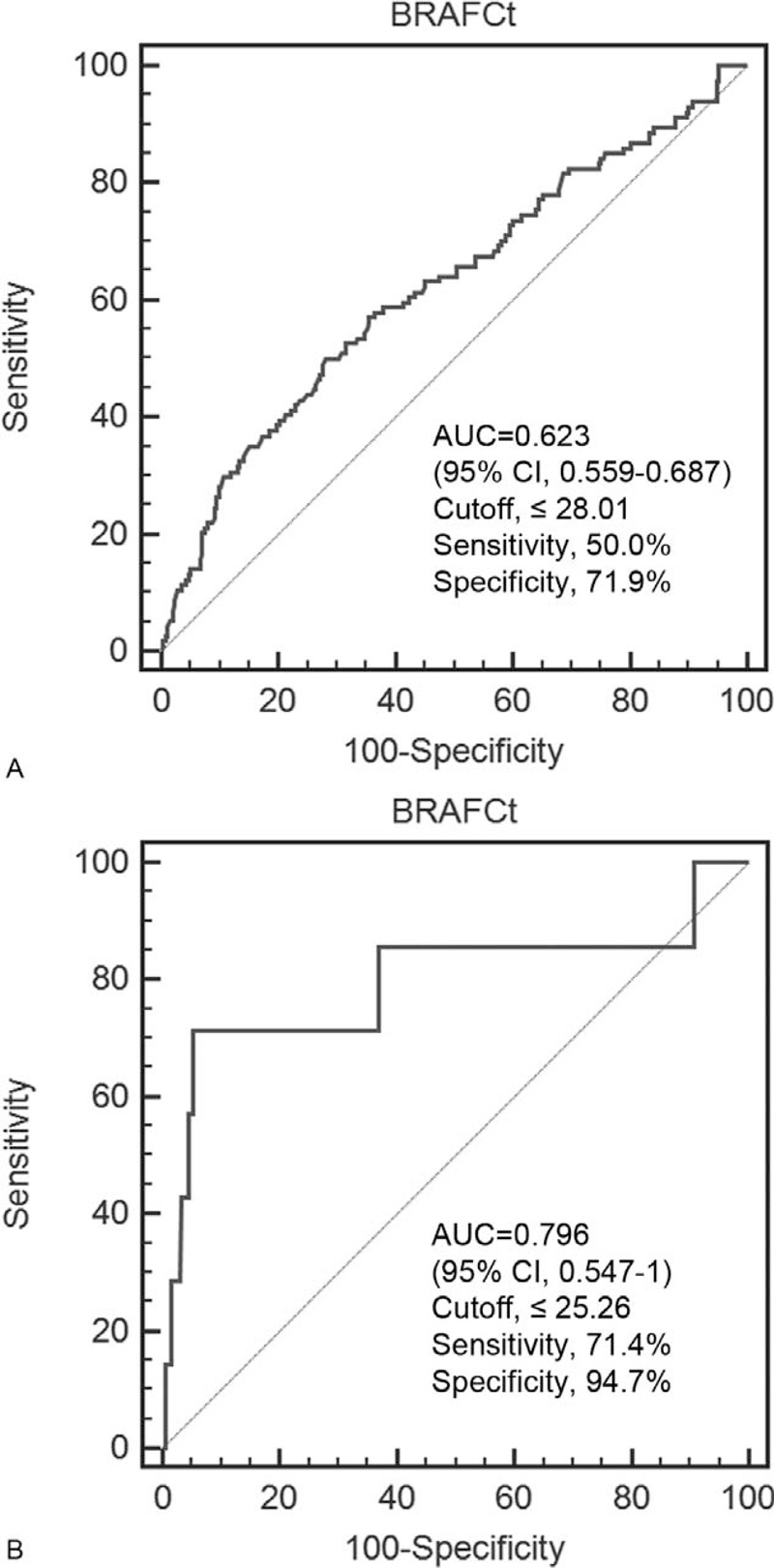
ROC curves of BRAF^V600E^ Ct values to distinguish patients with lymph node metastasis from patients without lymph node metastasis. (A) The use of a cut-off value of 28.01 revealed an area under the ROC curve (AUC) of 0.623 (95% CI 0.559–0.687) for the detection of central lymph node metastasis. (B) The use of a cut-off value of 25.26 revealed an AUC of 0.796 (95% CI 0.547–1) for the detection of lateral lymph node metastasis. CI = confidence interval, Ct = cycle threshold, ROC = receiver-operating characteristic.

The area under the ROC curve (AUC) for distinguishing central LNM from noncentral LNM groups was 0.623 (95% CI 0.559–0.687, *P* < 0.001) at a BRAF^V600E^ Ct cut-off value of 28.01 (sensitivity, 50.0%; specificity, 71.9%) (Figure 1A). The AUC for distinguishing lateral LNM from nonlateral LNM groups was 0.796 (95% CI 0.547–1, *P* = 0.02) at a BRAF^V600E^ Ct cut-off value of 25.26 (sensitivity, 71.4%; specificity, 94.7%) (Figure 1B).

## DISCUSSION

We found that the Ct value of the BRAF^V600E^ mutation was an independent factor for predicting central LNM in patients with PTMCs in a BRAF^V600E^-prevalent population. Because a lower Ct value corresponds to a higher BRAF^V600E^ mRNA level, we can assume that a negative association between Ct values and central LNM indicates a positive association with the quantitative expression of the BRAF^V600E^ mutation. Thus, a larger amount of RNA with the BRAF^V600E^ mutation expressed is associated with central LNM.^16,24^ Our results are the first to show that the Ct value, a quantitative measurement of the BRAF^V600E^ mutation, may be beneficial in the preoperative risk stratification of PTMC patients.

Although the association between the presence of the BRAF^V600E^ mutation and clinicopathologic prognostic factors of PTC has been extensively researched in the last decade, studies both on PTC and PTMC have shown variable results.^10–13,25,26^ In PTMC, the BRAF^V600E^ mutation has been associated with central and/or lateral LNM, multifocality, extrathyroidal invasion, and advanced tumor stage.^8,11,12,25,26^ However, other studies have reported the presence of the BRAF^V600E^ mutation in a significant percentage of PTMC patients without a significant association with poor prognostic factors.^10,13^ Such variable results have been similarly reported in Korean PTC patients, who have a much higher prevalence of the BRAF^V600E^ mutation than patients of different ethnicities and who live in an iodine-sufficient area.^10,16,26,27^ The controversial relationship between the presence of the BRAF^V600E^ mutation and prognostic factors representing tumor aggressiveness has been recently considered to be partly due to the heterogeneous distribution of the BRAF^V600E^ mutation within tumors.^14,15,17,18,28^ Furthermore, false-positive results for the BRAF^V600E^ mutation have been reported, especially when using highly sensitive analytic methods such as real-time PCR, and this may have contributed to such controversial results. Although a Ct cut-off value of 40 was reported to have a positive rate of 100% in our institution, nodules with Ct values ranging from 40 to 32.4 in this study may also include false-positives as the Ct cut-off value showing a specificity of 100% was 32.4.^20^ In this study, the BRAF^V600E^ mutation showed no significant association with variable clinicopathologic factors when qualitatively detected, which is consistent with several studies which have shown no association between the BRAF^V600E^ mutation status and prognostic factors for PTMC.^10,13^

Although Ct values were significantly lower in patients with lateral LNM, there was no significant association between Ct values and lateral LNM at multivariate analysis (*P* = 0.111). However, at ROC curve analysis of BRAF^V600E^ Ct values, the AUC for distinguishing patients with lateral LNM from those without lateral LNM was larger than that for distinguishing central LNM and noncentral LNM groups, with both higher sensitivity and specificity. These results may imply that whereas an association between BRAF^V600E^ Ct values and central LNM was identified in our study due to the sufficient number of events (n = 114), a possible association with lateral LNM may not have been identified with multivariate analysis due to the small number of lateral LNM events (n = 7). Further studies with a larger number of lateral LNM events are needed to evaluate the association between BRAF^V600E^ Ct values and lateral LNM in PTMC patients.

Previous reports based on surgical specimens showed no significant association between the allelic percentage of the BRAF^V600E^ mutation and LNM in patients with PTC.^17,18,28^ This may be attributed to differences in the study population, which showed more heterogeneity in tumor size and patient management. The mean tumor volume or tumor size varied from 14.9 mL and 2.2 cm and 80% of the patients underwent central neck dissection, with no relevant information presented in 1 study.^17,18,28^ These results that contrasted with our own suggest that the association between quantitative measurements of the BRAF^V600E^ mutation and LNM are more prominent or limited to small tumors such as microcarcinomas. Furthermore, microcarcinomas have been shown to be more heterogeneous with respect to the BRAF^V600E^ mutation when compared with larger tumors, and thus a quantitative expression of the BRAF^V600E^ mutation may be a more useful prognostic factor in this subgroup.^15^ In addition, because of the relatively high prevalence of the BRAF^V600E^ mutation in PTMCs (79.8%, 367 of 460), identifying an association between the quantitative expression of the BRAF^V600E^ mutation and LNM may have been easier in our study population.

A high percentage of BRAF^V600E^ alleles has been associated with larger tumor size, extrathyroidal extension, or recurrence in PTC, but previous studies were based on surgically resected tumor specimens and thus were not applicable in preoperative clinical settings.^17,18^ In this study, the BRAF^V600E^ Ct values were significantly correlated with tumor size in PTMCs, similar to previous studies that analyzed the percentage of mutant BRAF^V600E^ alleles or its relative expression in PTC.^16–18^

The role of routine prophylactic central LN dissection in the treatment of PTMC remains controversial. Currently, the American Thyroid Association only recommends central LN dissection in clinically involved neck LNs or in T3 or T4 tumors, and some authors suggest that prophylactic central LN dissection may increase the risk of complications and overall morbidity without improvement in survival.^29,30^ However, subclinical central LNM is frequent in PTMC and prophylactic central LN dissection may reduce short-term locoregional recurrence.^31^ Therefore, a more selective approach in the use of prophylactic central LN dissection may be more reasonable to avoid the minimal potential for morbidity.^32^ Because US shows low sensitivity in identifying central LNM, researchers have strived to find preoperative predictive factors for central LNM.^22,33,34^ With further validation with large sample studies, our results may also aid in the preoperative decision-making regarding surgical treatment.

Our study had several limitations. First, this was a retrospective study, and we included patients who underwent FNA and additional BRAF^V600E^ testing. Therefore, some selection bias is inevitable. Second, although the Ct value quantitatively reflects gene expression in real-time PCR, it is determined from a log-linear plot of the PCR signal versus the cycle number and thus, is not a linear term.^21^ For accurate relative quantification of real-time PCR data, comparisons relative to a reference gene are required. However, in real clinical practice, the routine acquisition of such data is both impractical and impossible. Furthermore, studies in other fields have directly utilized the Ct value as a relative measure of mRNA concentration levels.^24,35^ As real-time PCR is increasingly used in the detection of the BRAF^V600E^ mutation, our study demonstrates the possibility of utilizing a quantitative value which is easily attainable in actual clinical practice. Another limitation of our study was that the BRAF^V600E^ mutation analysis was performed with fine needle aspirates, and we did not evaluate possible contamination by nonthyroid cells such as lymphocytes. However, our study has clinical value in that it investigates the actual potential use of BRAF^V600E^ Ct values in preoperative decision-making for PTMC patients. Also, because only PTMCs were included, the majority of the tumor volume was more likely to be included when performing US-FNA with the freehand technique. Finally, Ct values were not statistically significant independent predictors of lateral LNM at multivariate analysis, even though the AUC for distinguishing patients with lateral LNM from those without lateral LNM was higher than that for distinguishing central LNM and noncentral LNM groups. Statistically, the limited number of lateral LNM cases may have resulted in a type-II error, which means that significant differences were not detected even with true differences existing between the groups. Therefore, further studies are needed to evaluate the real association between BRAF^V600E^ Ct values and lateral LNM in PTMC patients with a larger number of events.

In summary, this study demonstrates that real-time PCR Ct values for the BRAF^V600E^ mutation obtained from fine needle aspirates can be associated with central LNM in PTMC patients. Although BRAF^V600E^ Ct values did not reach statistical significance for predicting lateral LNM in our study, further validation through larger studies can be used to overcome any possible type-II errors. With more research, PCR Ct values for the BRAF^V600E^ mutation obtained from fine needle aspirates may have important implications for predicting both central and lateral LNM in patients with PTMCs.
